# Indications of Optical Coherence Tomography in Keratoplasties: Literature Review

**DOI:** 10.1155/2012/989063

**Published:** 2012-10-15

**Authors:** Thiago Trindade Nesi, Daniel Amorim Leite, Fábio Medina Rocha, Marco Antônio Tanure, Pedro Paulo Reis, Eduardo Büchele Rodrigues, Mauro Silveira de Queiroz Campos

**Affiliations:** ^1^Department of Ophthalmology, Cornea Unit, Federal University of Minas Gerais, São Geraldo Hospital, 190 Alfredo Balena Avenue, 30130-100 Belo Horizonte, MG, Brazil; ^2^BH Olhos Clinic, Cornea Unit, 1900 Bahia St, 30160-011 Belo Horizonte, MG, Brazil; ^3^Department of Ophthalmology, Vision Institute, UNIFESP, 821 Botucatu St, 04023-062 São Paulo, SP, Brazil

## Abstract

Optical coherence tomography (OCT) of the anterior segment, in particular corneal OCT, has become a reliable tool for the cornea specialist, as it provides the acquisition of digital images at high resolution with a noncontact technology. In penetrating or lamellar keratoplasties, OCT can be used to assess central corneal thickness and pachymetry maps, as well as precise measurements of deep stromal opacities, thereby guiding the surgeon to choose the best treatment option. OCT has also been used to evaluate the keratoplasty postoperative period, for early identification of possible complications, such as secondary glaucoma or donor disc detachments in endothelial keratoplasties. Intraoperatively, OCT can be used to assess stromal bed regularity and transparency in anterior lamellar surgeries, especially for those techniques in which a bare Descemet's membrane is the goal. The purpose of this paper is to review and discuss the role of OCT as a diagnostic tool in various types of keratoplasties.

## 1. Introduction

Initially developed in 1991 [1] and first used for imaging of the cornea and anterior segment of the human eye in 1994 [2], the optical coherence tomography (OCT) has already become a reliable tool for anterior segment propaedeutic. OCT uses low-coherence interferometry to produce a two-dimensional image of optical scattering from internal tissue microstructures. It has been developed for noninvasive and noncontact cross-sectional imaging in biological systems [1].

The original OCT technology has been reclassified as time-domain OCT (TD-OCT). More recently the Fourier-domain OCT (FD-OCT) was developed, also named spectral-domain OCT (SD-OCT), spectral OCT, high-definition OCT (HD-OCT), and frequency-domain OCT (FD-OCT). 

The TD-OCT is represented by Visante OCT (Carl Zeiss Meditec, Dublin, OH, USA) and Slit-lamp OCT (Heidelberg Engineering GmbH, Heidelberg, Germany). Both were developed for anterior segment imaging (AS-OCT), using 1310-nm wavelengths. This longer wavelength provides images with reduced scattering and less signal loss in opaque tissues, allowing deeper penetration of the limbus for the visualization of the scleral spur and angle recess, with a 15–20 *μ*m resolution. An advantage of these devices is a wider area of capture in a single image [3].

Originally designed for retinal imaging, the FD-OCT utilizes shorter wavelengths compared to TD-OCT (830-nm), which gives a better tissue resolution of 5 *μ*m, allowing visualization of details that was not possible with the older technology, however, with less penetration in opaque medias and narrow images. Examples of devices are the RTVue OCT (Optovue Inc., Fremont, CA, USA) and Cirrus OCT (Carl Zeiss Meditec, Dublin, OH, USA). While the former must be adapted with one of the two types of cameras for capturing cornea and anterior segment images [3], the current Cirrus SD-OCT device has inbuilt anterior segment imaging options without the need for modifications. 

Another difference between TD-OCT and FD-OCT is the speed of image capture, which can be 10 to 100 times faster with the latter, thus minimizing the impact of any eye movement on the exam results. In addition, the higher speed improves the quality of the images and gives a higher definition for using more scans in the same transverse localization.

In recent years, new techniques of keratoplasties have been developed, for example, femtosecond-assisted penetrating keratoplasty (FAPK), Descemet-stripping endothelial keratoplasty/Descemet-stripping automated endothelial keratoplasty (DSEK/DSAEK), Descemet-membrane endothelial keratoplasty (DMEK), and deep anterior lamellar keratoplasty (DALK). Advances have also been made in the evaluation of anterior segment structures with OCT, like the cornea. Many studies have related the numerous advantages in utilizing OCT, as it helps the surgeon to choose the best treatment option for a corneal disease. Deeper opacities require deeper lamellar techniques, or even a penetrating keratoplasty (PK). Furthermore, the postoperative followup period of keratoplasties has several OCT indications; especially in those cases where corneal edema makes anterior segment evaluation impractical, and for children submitted to PKs [4], since it is a noninvasive technique. OCT is also a valuable intraoperative tool [5, 6]. Cases of lamellar keratoplasties, in which we search for the most regular interface to achieve the best visual results, are a particular indication of its intraoperative use. 

The objective of this paper is to review the main indications and findings of corneal OCT in eyes submitted to penetrating and lamellar keratoplasties.

## 2. OCT in Penetrating Keratoplasty (PK)

Considered the gold-standard technique in keratoplasty until a decade ago, the PK is now being substituted for lamellar techniques, seeking the maintenance of healthy tissues, and the change only of the diseased layer of the cornea. However, the use of femtosecond laser in the ophthalmology allowed the PKs to have a considerable advantage in their technique and outcomes. With this new technology, it is possible to perform precise cuts in different shapes that optimize the wound alignment, improving its biomechanics, and possibly reducing the postoperative astigmatism [7]. Some software products were developed in order to perform a number of cut patterns, such as the Intralase (AMO, Inc, Santa Ana, CA, USA), the Technolas laser (Technolas PerfectVision, Heidelberg, Germany), the Femto LDV (Ziemer Ophthalmic Systems AG, Port, Switzerland), and the VisuMax laser (Carl Zeiss Meditec AG, Jena, Germany). The most used cut configurations are zigzag, mushroom, top-hat, Christmas tree, and others [8]. These configurations create a larger contact area in the graft-host junction and are more stable when compared with the traditional manual vertical trephination [8, 9].

**Figure 1:**
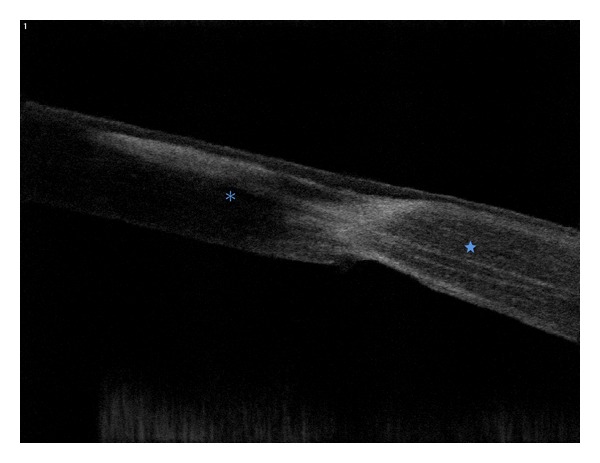
Horizontal section with RTVue OCT of a patient who underwent traditional PK three years ago. Note the malapposition between the donor (asterisk) and the host cornea (star).

It is possible with OCT to study the wound anatomy, giving another perspective of manual PKs, as shown in Figure 1. The images can clearly show the graft-host junction malapposition in the majority of cases, helping to better understand suboptimal postoperative results, despite normal topographies, as shown by Kaiserman and coworkers [10]. This study evidenced that larger malappositions are associated with higher postoperative astigmatism, myopia, and intraocular pressure (IOP). Steeper grafts and optical tilt aberrations correlate with thinner graft-host touch, and are supposedly less stable.

It is possible to evaluate the configuration details of the femtosecond laser cuts with OCT, confirming in most cases a stronger and faster wound apposition when compared with the manual vertical trephination, and further to show a better alignment between the donor tissue and the host cornea [11, 12]. A previous study [13] compared the resistance of the wounds in different techniques: traditional PK, FAPK top-hat with suture, and FAPK top-hat with suture and fibrin glue as an adjunct (groups 1, 2, and 3, resp.). The authors, despite executing only a laboratory experiment, showed that group 3 had a resistance of wound burst pressure much higher than the others (*P* < 0,0001). Nevertheless, the induced astigmatism was slightly higher than in groups 1 and 2, but without a statistical significance. We should consider that the best way to compare the real induced astigmatism is after the suture removal, what was not evaluated in this study.

OCT also plays a role in evaluating the most common postoperative complications for PKs. Studies utilize OCT in cases where Descemet's membrane was not fully excised and in cases of secondary postoperative glaucoma. These cases can generate corneal edema or other opacities that would hinder the proper evaluation of the anterior segment anatomy with slit-lamp examination, but where it would be possible to visualize through OCT [14].

Some researches with OCT are being developed, like its use at eye banks to screen corneas that have already been submitted to refractive procedures, which are sometimes impossible to see with biomicroscopy, and are a counter-indication for usage in a PK. A study demonstrated that OCT has some advantages over corneal topography, which is another device to identify corneas that have already gone through any kind of refractive surgery. For example, OCT can capture images with the tissue immersed in preservation medium in a sterile view chamber, avoiding air exposure, and reducing the risk of contamination, unlike topography [15].

## 3. OCT in Anterior Lamellar Keratoplasty (ALK)

The ALK has its principal indications in cases of stromal scars after infectious keratitis, trauma, stromal dystrophies opacities, and in ectasia cases, such as keratoconus [16]. In the latter cases, it is advisable to perform the DALK technique, in order to leave a bare Descemet's membrane. These patients have an unaltered endothelium cell layer, and do not need to be exchanged, like PK cases [16].

For this reason, ALK has some advantages over PK, like performing a close-chamber surgery, rarely presents with endothelial failure (endothelial cell loss occurs only during the procedure 8 to 15%), faster recovery, absent of endothelial rejection, greater trauma resistance, and others [16, 17].

**Figure 2:**
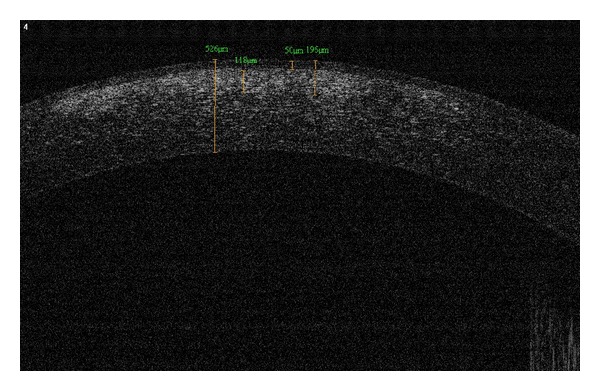
A patient with permanent opacities in the visual axis due to an episode of Adenoviral conjunctivitis. Note the presence of deep opacities (196 *μ*m with corneal epithelium).

Depending on the indication, the ALKs can have different thicknesses. In cases of superficial opacities (Figure 2), basal membrane or stromal dystrophies [18], it is not mandatory to exchange all the recipient stroma, which maintains a safe boundary and results in a safer surgery, as the Descemet's membrane is not exposed.

On the other hand, with deeper opacities or ectasia cases; such as keratoconus, the ideal procedure is to separate all the deep stromal layers from the Descemet's membrane [18], leaving it practically bare before suturing the donor cornea disc. There are a few techniques that help the surgeon to achieve this extremely delicate step, like the Big Bubble technique, described by Anwar and Teichmann in 2002 [19]. Some innovations have been created, such as the use of enzymatic digestion of the corneal stroma and extracellular matrix that facilitates the separation between Descemet's membrane and deep stroma [20]. The OCT plays an important role and has advantages over Confocal microscopy and immersion high-frequency ultrasound biomicroscopy (UBM) in cases where the stromal opacities are deeper, since it can evaluate the thickness of the opacity with extreme precision, helping the surgeon to choose a better strategy for each case [16, 18, 21].

**Figure 3:**
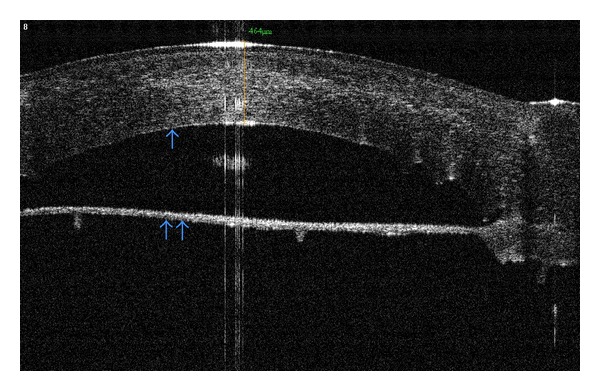
A case of DALK presenting persistent Descemet's membrane detachment (double arrow). The surgeon noted extended detachment during the Big-Bubble procedure, which was not resolved after air injection into anterior chamber. Note the remarkable pachymetry of the donor graft. The Descemet's membrane anCCd endothelium of the donor were not stripped (single arrow). Three-month followup with RTVue OCT.

A very important factor in postoperative success is the interface regularity, as well as the recipient stroma thickness [22]. DALK surgeries that reach Descemet's membrane have improved outcomes, even comparable to PK visual results in some studies [23, 24]. Others describe the intraoperative use of OCT [6] in order to analyze the interface (showing thicker or irregular stromal areas, allowing the surgeon to better evaluate the recipient bed), and its increased transparency [22]. OCT also has indications on the postoperative followup of ALKs, helping screen for possible complications, for example, double or triple-anterior chamber [25], Descemet's membrane detachment [26] (Figure 3), and interface keratitis [27].

## 4. OCT in Endothelial Keratoplasty (EK)

The modern EK procedure was first described in 1998 by Melles et al. [28], presenting some advantages over PK in selected cases, such as endothelial dystrophies, pseudophakic or aphakic bullous keratopathy or other endothelial dysfunction. Regular topography, maintenance of the ocular surface, faster recovery, and a more stable wound are some known advantages [29].

In light of these advantages, EK is also an indication in failed PKs, considering that a new PK has disadvantages of longer visual recovery, suture problems and lower success rates, along with rejection risks [30]. This study describes some surgical strategies to enhance graft apposition, for example, stripping only the Descemet's membrane inside the full-thickness graft to avoid any manipulation at the posterior graft-host junction. The authors also recommend inserting a smaller donor disc, in order to improve adherence. OCT, they say, is particularly valuable to guide the choice of the graft diameter, avoiding DSAEK graft edge lift, and reducing dislocations.

Complications of this technique are well studied, and using OCT in these cases, particularly in edematous corneas, is very helpful in order to evaluate the anterior segment anatomy and possible complications that might appear during the postoperative period. Interface opacities [31], persistent lamellar fluid [32], epithelial ingrowth [33] and principally donor disc dislocation [34–36], are some described intercurrences.

Another potential complication is the elevation of IOP after the procedure. Some studies relate pupillry block by the air bubble left at the end of the surgery [37], in search of optimal apposition between the donor tissue and the recipient stroma. In other cases, the air bubble can migrate to the posterior chamber, pushing the iris root and raising the IOP [38]. OCT has an important role in many cases where it is difficult to evaluate through an edematous cornea and to visualize the anterior chamber angle, as well as possible anterior synechiae.

**Figure 4:**
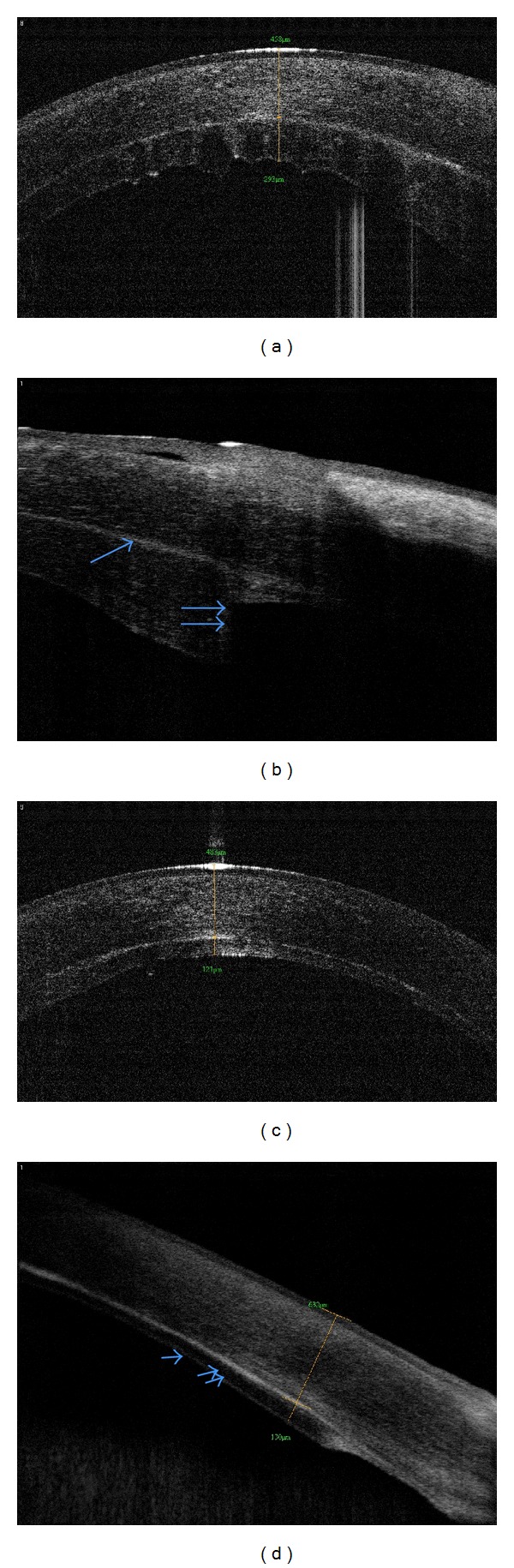
Patient who underwent DSEK in both eyes. First surgery was performed in the right eye, and a regraft (a) was needed due to endothelial failure two months later. (b) Note the graft edge irregularity (single arrow = graft-host interface; double arrow = donor edge). The left eye had an uneventful surgery (c), with optimal apposition (d) of the donor disc (single arrow = donor endothelium; double arrow = donor stroma).

Its use is also indicated in the followup of corneal deturgescence [39], as well as in the evaluation of the donor disc and recipient stroma adhesion and apposition [40]. Another described indication of OCT for EKs is the study of donor tissue thickness and its regularity [41, 42] (Figure 4), which may influence the final visual acuity and refractional changes after surgery. Studies show a relationship between thicker grafts and limitations on the visual improvement, as well as hyperopic shifts caused by the effect of a minus lens inside the eye and also by reducing the posterior curve of the cornea, both acting in this refractional change [43]. In a recent published paper [44], Higashiura and coworkers analyzed corneas submitted to DSAEK by topographic characteristics of anterior host part and the posterior graft using OCT-based corneal topography. This newly introduced tool might also be useful in determining the factors associated with optical quality of the cornea following DSAEK.

## 5. Conclusion

Various keratoplasty techniques are available and in constant development. It is up to the surgeon to choose the best one for each case, while considering the pathology and which corneal layer (or layers) should be exchanged. This produces faster visual recovery, enhanced visual results, and a more satisfied patient. OCT is established as a valuable tool to assess corneal pathologies, and it has been widely used in cases of keratoplasties to provide high resolution images that help the indication of one technique or another. In addition, OCT provides important information about the anterior segment anatomy in the postoperative period, particularly in cases which are difficult due to optical irregularities, such as corneal edema.
